# A Rationally Designed ICAM1 Antibody Drug Conjugate for Pancreatic Cancer

**DOI:** 10.1002/advs.202002852

**Published:** 2020-11-03

**Authors:** Jing Huang, Agoston T. Agoston, Peng Guo, Marsha A. Moses

**Affiliations:** ^1^ Vascular Biology Program Boston Children's Hospital Boston MA 02115 USA; ^2^ Department of Surgery Boston Children's Hospital and Harvard Medical School Boston MA 02115 USA; ^3^ Department of Pathology Brigham and Women's Hospital Harvard Medical School Boston MA 02115 USA

**Keywords:** antibody drug conjugates, ICAM1, magnetic resonance imaging, pancreatic cancer, targeted therapy

## Abstract

Outcomes for pancreatic cancer (PC) patients remain strikingly poor with a 5‐year survival of less than 8% due to the lack of effective treatment modalities. Here, a novel precision medicine approach for PC treatment is developed, which is composed of a rationally designed tumor‐targeting ICAM1 antibody‐drug conjugate (ADC) with optimized chemical linker and cytotoxic payload, complemented with a magnetic resonance imaging (MRI)‐based molecular imaging approach to noninvasively evaluate the efficiency of ICAM1 ADC therapy. It is shown that ICAM1 is differentially overexpressed on the surface of human PC cells with restricted expression in normal tissues, enabling ICAM1 antibody to selectively recognize and target PC tumors in vivo. It is further demonstrated that the developed ICAM1 ADC induces potent and durable tumor regression in an orthotopic PC mouse model. To build a precision medicine, an MRI‐based molecular imaging approach is developed that noninvasively maps the tumoral ICAM1 expression that can be potentially used to identify ICAM1‐overexpressing PC patients. Collectively, this study establishes a strong foundation for the development of a promising ADC to address the critical need in the PC patient care.

## Graphical abstract

A novel precision medicine approach for pancreatic cancer (PC) treatment is developed. It is composed of a rationally designed tumor‐targeting ICAM1 antibody‐drug conjugate (ADC) with an optimized chemical linker and cytotoxic payload, complemented by a magnetic resonance imaging (MRI)‐based molecular imaging approach to noninvasively evaluate the efficiency of ICAM1‐targeting immunotherapy.

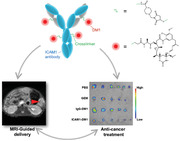

## 1 Introduction

Pancreatic cancer (PC) remains among the most lethal diseases today, projected to account for ≈56 770 deaths in the United States in 2019 alone, representing 7% of all cancer mortality.^[^ ^1^ ^]^ The prognosis for PC patients is poor with a 5‐year survival of 9.3% despite the recent applications of immunotherapy and nanomedicine therapy.^[^ ^2^ ^]^ For instance, immune checkpoint blockade therapies including cytotoxic T‐lymphocyte‐associated antigen 4 inhibitor or programmed death‐ligand 1 antibodies have yet to show successful clinical efficacy in treating PC patients.^[^ ^3^, ^4^ ^]^ Innovative nanomedicine formulations (e.g., EphA2‐targeted liposomal docetaxel) also failed to provide significant clinical benefit in treating advanced PC.^[^ ^5^ ^]^ These disappointing results are largely due to the tumor microenvironment (TME) of PC tumors which is characterized by desmoplastic stroma and poor vascularization. Such a TME creates physical barriers that prevent T‐cells or nanomedicines from efficiently infiltrating tumors and directly interacting with PC cells, leading to unfavorable efficacies.^[^ ^6^ ^]^ There is, therefore, a critical need to develop novel targeted therapeutics that can better access PC tumors while maintaining potent tumor‐specific efficacy.

Antibody‐drug conjugates (ADCs) are a rapidly growing class of targeted therapeutics that have shown promising clinical efficacy against several types of cancers including aggressive solid tumors such as breast cancer that respond poorly to T‐cell immunotherapy.^[^ ^7^, ^8^, ^9^ ^]^ Unlike conventional chemotherapeutics, ADCs utilize chemical linkers to conjugate cytotoxic drugs to tumor‐homing antibodies which are capable of selectively targeting the tumor while sparing normal tissues. They accomplish this via recognizing tumor surface antigens, subsequently internalizing and delivering the cytotoxic drugs into targeted tumor cells. Compared to T‐cell immunotherapy (e.g., chimeric antigen receptor‐T cell (CAR‐T) or immune checkpoint blockade) or nanomedicines (e.g., liposomes), ADCs feature superior tumor tissue penetration due to their ultrasmall size (<10 nm), creating an attractive opportunity to increase drug delivery into stroma‐dense PC tumors.

However, a major hurdle in developing PC‐targeted ADC drugs is identifying suitable ADC targets that effectively distinguish between PC and normal tissues. To meet the safety and efficacy criteria for ADCs, such targets should be abundantly presented on the cell surface of PC tumors with undetectable levels in normal tissues, making it exclusively accessible to the tumor‐homing antibody of the ADC. These characteristics are also required to facilitate a rapid and robust cell internalization of cytotoxic payloads conjugated on the ADCs. Though several conventional cancer targets (e.g., EGFR, EphA2, and mesothelin) have been suggested for PC‐targeted therapy,^[^ ^10^, ^11^, ^12^ ^]^ their cell surface abundance have not been validated/compared systematically and quantitatively. We hypothesized that performing such unbiased and quantitative screening of cell surface proteins would lead to the discovery of effective and specific PC targets and to the development of PC‐targeted ADCs.

ICAM1(CD54) is a transmembrane glycoprotein of the immunoglobulin superfamily which is aberrantly overexpressed in multiple types of cancers (e.g., PC, triple negative breast cancer, melanoma, and thyroid cancer) and is frequently associated with an aggressive phenotype and poor prognosis.^[^ ^13^, ^14^, ^15^, ^16^, ^17^, ^18^, ^19^ ^]^ In PC, ICAM1 is directly induced on pancreatic acinar cells by KRAS^G12D^, the most common oncogenic mutation in 70–95% PC patients that drives the formation of pancreatic neoplastic lesions, leading to PC tumor initiation.^[^ ^20^ ^]^ The present studies describe the identification and application of ICAM1 as a potential PC target based on our unbiased and quantitative screening algorithm. We have developed a proof‐of‐principle ICAM1 ADC that induces potent and durable PC tumor regression in vivo. To build a precision medicine, we further developed a noninvasive MRI approach to identify ICAM1‐expressing tumors suitable for ICAM1‐targeting ADC therapy. Taken together, these studies demonstrate the development of a novel ICAM1 ADC that can be successfully utilized to stratify, select, treat, and monitor those patients who might best benefit from this precision medicine approach to PC therapy.

## 2 Results

### 2.1 Identification of PC Cell Surface Target

**Figure 1:**
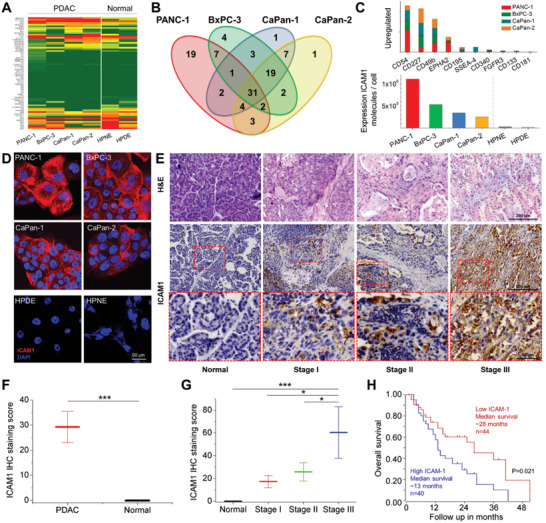
Differential overexpression of ICAM1 in human PC tissues and cells. A) Heatmap of membrane proteins expression in human PC cells, compared with normal pancreatic epithelial cells. B) Venn diagram showing the overlaps between the selected target sets for PC cells. C) Top 10 upregulated surface proteins in PC cells and ICAM1 expression level in human PC cell lines. D) IF staining of ICAM1 in human PC and normal pancreatic epithelial cells. E) Representative images of IHC staining of ICAM1 in human PC tumor tissues at different stages and normal pancreas tissues. F) Comparison of ICAM1 IHC staining score between PC (*N* = 80) and normal (*N* = 20) tissues. G) Pathological scores for tumor microarrays correlated with TNM stages (Normal: *N* = 20, Stage I: *N* = 22, Stage II: *N* = 41, Stage III: *N* = 15). H) Kaplan–Meier analysis of overall survival of PC patients (Low ICAM1: *N* = 44, High ICAM1: *N* = 40) according to different ICAM1 levels. *p < 0.05, **p < 0.01, ***p < 0.001.

To identify an optimal cell surface target that can distinguish malignant PC tumors from normal tissues, we utilized our platform for rationally designed cell surface protein target discovery ^[^ ^21^, ^22^, ^23^ ^]^ (**Figure** **1**A). We first performed an unbiased and quantitative screening of a panel of cancer‐related surface antigens in four established human PC cell lines (PANC‐1, BxPC‐3, Capan‐1, and Capan‐2) in comparison with two normal human pancreatic duct epithelial cells (HPDE and HPNE) as normal controls (Figure 1B). Thirty‐one candidates were found to be commonly overexpressed in all four PC cell lines and were selected for further evaluation. By comparing their levels in human PC cells and normal pancreatic cells, ICAM1 emerged as the most overexpressed PC target among the top ten candidates with almost no expression in non‐neoplastic HPNE and HPDE cells (Figure 1C). Notably, the cell surface density of ICAM1 ranges from 3 × 10^5^ to 1 × 10^6^ molecules per cell on the four PC cell lines, significantly higher than that of established PC targets (e.g., EGFR, MUC1, or EphA2). Additionally, ICAM1 is ubiquitously overexpressed across all tested human PC cell lines. We further confirmed the overexpression of ICAM1 is human PC cells using immunofluorescent (IF) staining. ICAM1 was predominantly expressed on the plasma membranes of four PC cell lines (PANC‐1, BxPC‐3, Capan‐1, and Capan‐2) but absent on normal HNPE and HPNE cells (Figure 1D). This selectively strong cell surface expression of ICAM1 on human PC cells makes it readily assessable for ICAM1‐targeting therapeutics (e.g., ADCs).

To investigate whether high ICAM1 expression is a clinically relevant finding in human PC, we conducted immunohistochemical (IHC) staining of ICAM1 in 80 human PC tumor tissues and 20 normal pancreas tissues. In Figure 1E,F, ICAM1 was consistently overexpressed on the plasma membrane and in the cytoplasm of PC cells from tumor tissues at different disease stages while being absent in the normal human pancreas tissues. The extent of staining and the pathological scores of ICAM1 showed that ICAM1 level was positively correlated to disease TNM stages (Figure 1G). We also carefully evaluated the on‐target, off‐tumor sites for ICAM1‐targeting therapeutics in normal tissues. We examined the protein levels of ICAM1 in a comprehensive cohort of 45 normal human organs by querying the Human Protein Atlas database (https://www.proteinatlas.org). We found that ICAM1 expression is absent in most normal tissues by IHC analysis with only 4% (2/45, lung and kidney) of normal tissues showing high positive staining of ICAM1, respectively.

We also investigated the impact of ICAM1 overexpression on clinical outcomes of PC patients by querying the R2: Genomics Analysis and Visualization Platform database (https://hgserver1.amc.nl/, Datasheet: Mixed Pancreas Tumor‐Zhang). The overall survival of PC patients with high ICAM1 expression was significantly worse than those with low ICAM1 expression (Figure 1H, *p* = 0.021, log–rank test), suggesting that ICAM1 may also serve as a clinical biomarker of poor prognosis in PC patients.

### 2.2 Recognition and Targeting PC Tumors by ICAM1 Antibody

**Figure 2:**
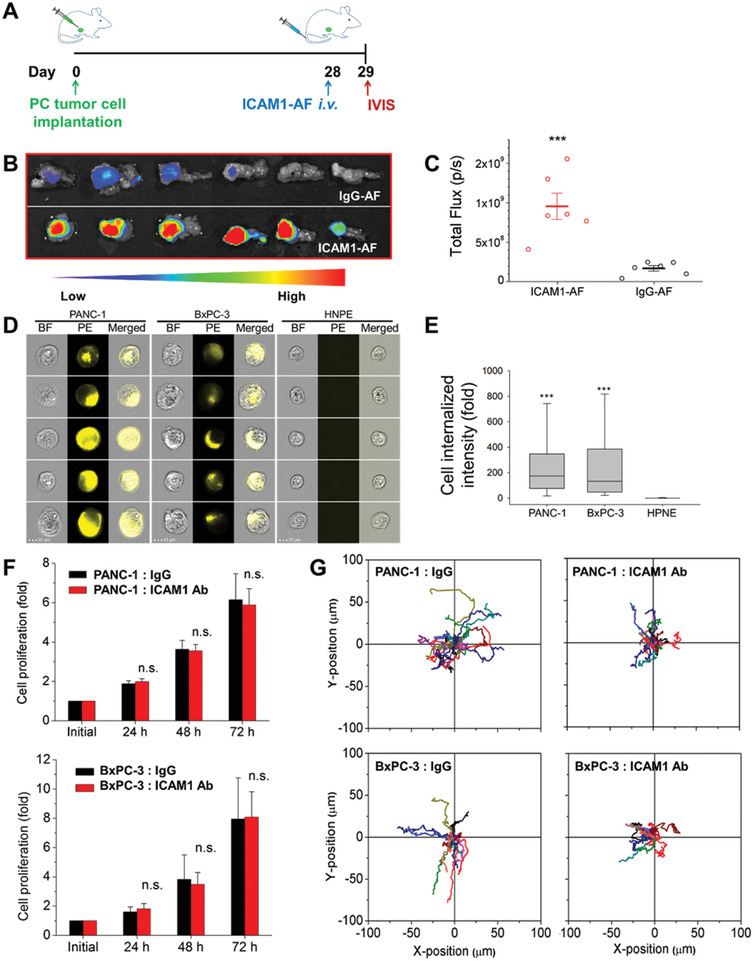
Specific recognition and targeting of PC tumor by ICAM1 antibody (ICAM1 Ab). A) Schematic diagram of the orthotopic PC model injected PANC1‐LUC at Day 0, receiving ICAM1‐AF or IgG‐AF at 28 days post tumor cell injection (*N* = 6 per group) with fluorescence imaging performed at Day 29. B) Ex vivo fluorescence imaging of PC tumors with surrounding normal pancreas tissues. C) Corresponding quantification of fluorescence intensity in tumors. D) Representative imaging flow cytometry images showing the PC‐specific internalization of ICAM1 Ab in PANC‐1, BxPC‐3 and HPNE cells. E) Signal intensity analysis for ICAM1 antibody‐mediated cell internalization (*N* = 10 000). F) Cell proliferation of PANC‐1 and BxPC‐3 with treatment of ICAM1 Ab or IgG. G) Cell motion trajectories showing the response of PANC‐1 and BxPC‐3 after 24 h treatment of ICAM1 Ab. *p < 0.05, **p < 0.01, ***p < 0.001.

To assess ICAM1 as a potential ADC target, we first determined the in vivo tumor‐specificity of ICAM1 antibody in an orthotopic PC tumor model (**Figure** **2**A). We fluorescently labeled ICAM1 monoclonal antibodies with AF‐647, a red fluorescent dye, (ICAM1‐AF) and intravenously injected them into PANC‐1 tumor‐bearing mice. AF‐647 labeled IgG (IgG‐AF) was used as a nontargeting control. Due to the fact that in vivo fluorescent signals are interfered with the intraperitoneal location of orthotopic PC tumors and abdominal skin absorption, we euthanized the animals at 24 h postinjection and excised PC tumors and their surrounding pancreatic tissues and then performed ex vivo fluorescent imaging to determine the tumoral accumulation of ICAM1‐AF antibodies.^[^ ^24^, ^25^ ^]^ As observed in Figure 2B, ICAM1 antibody selectively recognized and targeted orthotopic PC tumors with high affinity compared with nontargeting IgG controls. Notably, normal pancreatic tissues adjacent to PC tumors were not targeted by ICAM1 antibody, further confirming its PC tumor‐specificity. Quantified fluorescent signals (Figure 2C) confirmed that the tumoral accumulation of ICAM1 antibody is significantly higher (approximately sixfold) than that of nontargeting IgG‐AF after only one single dose of tail‐vein administration. These in vivo findings strongly support the development of ICAM1 antibody‐based therapeutics for PC‐targeted therapy.

Given that cell entry activity is a critical factor in ADC design,^[^ ^26^ ^]^ we investigated the cell internalization of ICAM1 antibodies in human PC cells using an imaging flow cytometry assay. As shown in Figure 2D, phycoerythrin (PE)‐conjugated ICAM1 antibody was robustly internalized by both PANC‐1 and BxPC‐3 cells via ICAM1 antigen‐mediated endocytosis, whereas almost no PE‐ICAM1 antibodies were internalized by normal HPNE cells due to the significant lack of ICAM1 antigen expression. The amount of PE‐ICAM1 antibody internalized by human PC cells was ≈300‐fold higher than that of HPNE cells (Figure 2E).

We next investigated the therapeutic consequences of blocking ICAM1 signaling cascades in human PC cells using its neutralizing antibody. In Figure 2F, treatment with ICAM1 neutralizing antibody (2 µg mL^−1^) did not obviously alter PANC‐1 or BxPC‐3 cell proliferation. However, ICAM1 neutralizing antibodies potently inhibited PANC‐1 and BxPC‐3 cell migration by 39% and 44%, respectively in comparison with IgG controls (Figure 2G). These findings indicate that this ICAM1 neutralizing antibody may not only serve as a PC tumor‐targeting ligand, but that it can also hinder disease progression by blocking ICAM1 signaling cascades.

### 2.3 Rational Design of ICAM1 Antibody‐Drug Conjugates

**Figure 3:**
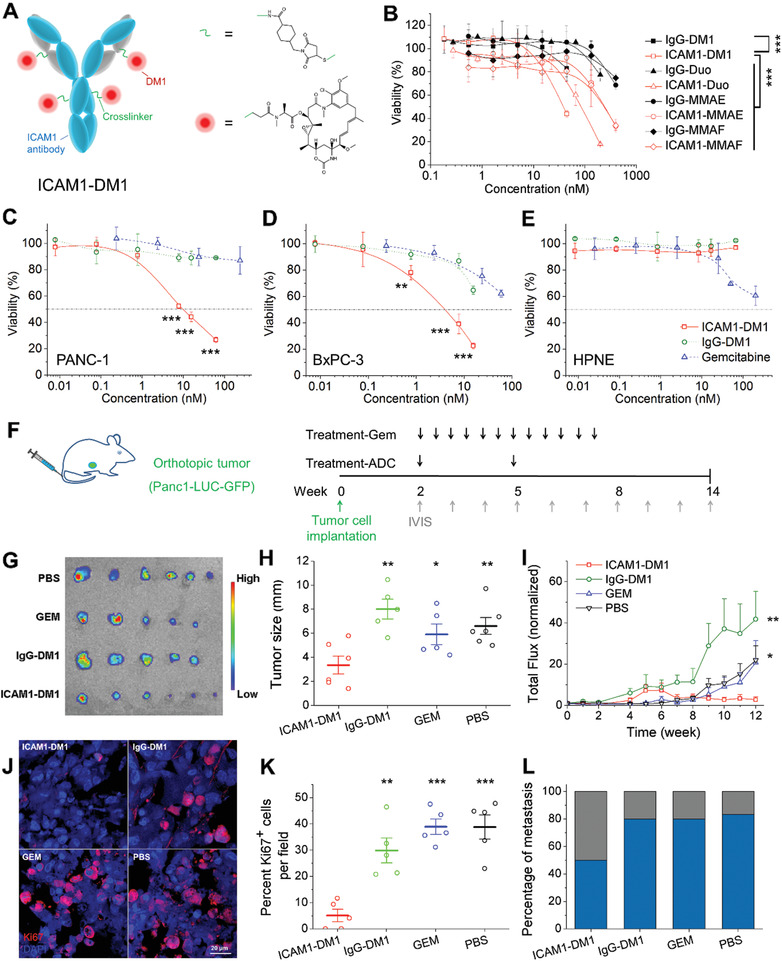
Selective ablation of PC cells by ICAM1‐DM1. A) Schematic illustration of ICAM1‐DM1 ADC. B) Screening of cytotoxic payload with different linker that conjugated to ICAM1 Ab. C–E) Cell viability assays to measure the anti‐tumor activity of ICAM1‐DM1 to C) PANC‐1 and D) BxPC‐3, and E) normal HPNE, compared with IgG‐DM1 and GEM. F) Schematic diagram of the orthotopic PC model injected PANC1‐LUC‐GFP at Week 0, receiving ICAM1‐DM1, IgG‐DM, gemcitabine or PBS at 2 weeks post tumor inoculation (*N* = 5,6 per group). IVIS was performed every week. G) Ex vivo fluorescence imaging of GFP‐expressing PC tumors and H) corresponding tumor diameters. I) Total flux of bioluminescence of the tumors in different treatment groups. J) Representative immunofluorescent staining of Ki67 (cell proliferation marker) in tumors of different treatment groups. K) Corresponding quantification of Ki67^+^ cell percentage. L) Percentage of metastasis (mice with metastasis/total mouse number) in different treatment groups. *p < 0.05, **p < 0.01, ***p < 0.001.

With the goal of leveraging our ICAM1 target to develop a novel PC therapy, we engineered a rationally designed ICAM1 ADC as a proof‐of‐principle PC‐targeted therapeutic(**Figure** **3**A). Given that the chemical linker and cytotoxic payload of ADC substantially affect the efficacy of ADC, our first step was to select the optimal ADC formulation for PC treatment using an unbiased and quantitative screening approach. We engineered a series of ICAM1 ADCs using four clinically tested ADC linkers and cytotoxic payloads (SMCC‐DM1, Vc‐MMAE, Mc‐MMAF, Duocarmycin) at equivalent drug‐to‐antibody ratio (DAR) of 1 and compared their cytotoxicity against human PC cells in comparison with nontargeting IgG ADC controls. As shown in Figure 3B, ICAM1‐SMCC‐DM1 showed the lowest IC50 (38.1 × 10^−9^ m) among four tested ADC formulations (other IC50s: 83.9 × 10^−9^ to 240.4 × 10^−9^ m) in treating PANC‐1 cells. It is noteworthy that the IC50 of ICAM1‐SMCC‐DM1 is over 2000‐fold lower than gemcitabine (GEM) (89.1 × 10^−6^ m), the current first‐line chemotherapeutic for PDAC therapy. SMCC‐DM1 is a clinically validated ADC formulation consisting of a non‐cleavable chemical linker and a potent microtubule inhibitor, Mertansine (DM1).^[^ ^7^ ^]^ We therefore selected SMCC‐DM1 as our optimized ADC formulation and subsequently synthesized ICAM1‐SMCC‐DM1 (ICAM1‐DM1) as the optimized ICAM1 ADC for PC‐targeted therapy. IgG‐SMCC‐DM1 (IgG‐DM1) was also prepared under the same experimental conditions as a nontargeting control. The DARs for ICAM1‐DM1 and IgG‐DM1 were controlled by the input amounts of DM1 and the antibodies and achieved 3.8 for ICAM1‐DM1 and 3.9 for IgG‐DM1 as determined using an UV/vis spectroscopy assay.^[^ ^27^, ^28^ ^]^

### 2.4 Selective Ablation of PC cells by ICAM1‐DM1

We determined the PC‐specific cytotoxicity of ICAM1‐DM1 in two widely used human PC cell lines, PANC‐1 (KRAS^G12D^ mutant) and BxPC‐3 (KRAS wildtype) along with normal HPNE cells (Figure 3C–E). The first‐line chemodrug GEM and nontargeting IgG‐DM1 were used as controls. As observed in Figure 3D,E, ICAM1‐DM1 showed potent cytotoxicity against PANC‐1 and BxPC‐3 cells. The IC50 of ICAM1‐DM1 was determined to be 9.8 × 10^−9^ m for PANC‐1 and 4.0 × 10^−9^ m for BxPC‐3, significantly lower than those of GEM and IgG‐DM1 (30 × 10^−9^ m to 88 × 10^−6^ m). Moreover, ICAM1‐DM1 showed no cytotoxicity in normal HPNE cells due to their lack of expression of ICAM1 (Figure 3E). These in vitro results strongly support the evaluation of the anti‐tumor activity of ICAM1‐DM1 in the in vivo setting of PC models.

We examined the anti‐tumor activity of ICAM1‐DM1 in suppressing orthotopic PC tumor growth in vivo (Figure 3F). ICAM1‐DM1 or IgG‐DM1 (non‐targeting control) was i.v. administered to PANC‐1‐Luc tumor‐bearing mice at 15 mg kg^−1^ every 3 weeks. This PANC‐1 model was selected because it is a well‐established PC model with the oncogenic KRAS^G12D^ mutation.^[^ ^29^, ^30^ ^]^ In comparison, as a control, GEM was weekly i.v. administered at a dose of 5 mg kg^−1^ due to its short circulation half‐life (0.28 h).^[^ ^31^ ^]^ After two ADC injections, the ICAM1‐DM1‐treated group exhibited a potent and durable tumor regression compared to other groups (Figure 3G–H). The quantified tumor mass showed that ICAM1‐DM1 significantly reduced PC tumor growth in comparison with the phosphate‐buffered saline (PBS) (sham) group (Figure 3I). We further examined The mechanism of ICAM1‐DM1 induced toxicity by measuring cell proliferation marker Ki67 expression in the PC tumor tissues. As observed in Figure 3J,K, the Ki67‐positive cell population in ICAM1‐DM1‐treated group was significantly reduced compared with the other groups, contributing to the potent and persistent tumor suppression. Importantly, this potent anti‐tumor activity of ICAM1‐DM1 also effectively inhibited spontaneous PC metastasis to normal organs including lung, liver, and spleen (Figure 3L; Figure S1, Supporting Information). Notably, there was no evidence of histopathological damage to the normal vital organs collected from the ICAM1‐DM1 treated group.

### 2.5 Noninvasive Evaluation of Tumoral ICAM1 Expression by MRI

**Figure 4:**
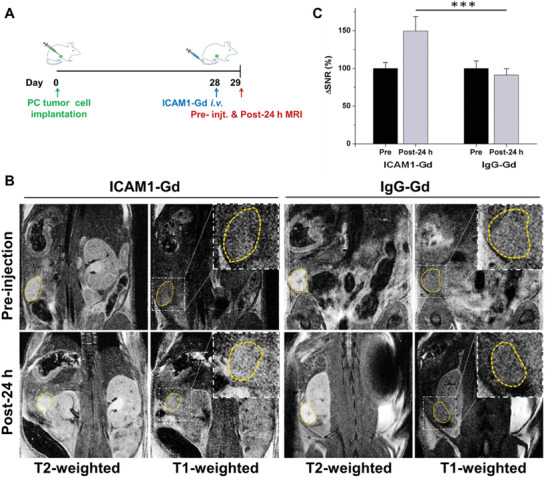
Assessment of ICAM1‐expressing PC tumors by noninvasive MRI. A) Schematic diagram of the orthotopic PC model injected PANC1‐LUC at Day 0, receiving ICAM1‐Gd or IgG‐Gd at 28 days post tumor inoculation (*N* = 3 per group) with MRI performed at Day29. B) Representative in vivo T1‐ and T2‐weighted MR images of mice bearing orthotopic PC tumors received ICAM1‐Gd and IgG‐Gd. Tumor was circled and magnified in the insets. C) Quantitative changes of MRI signal‐to‐noise ratio in PC tumors. Bar graphs are shown as mean ±SD. *p < 0.05, **p < 0.01, ***p < 0.001.

To build a precision medicine, we developed a MRI‐based molecular imaging approach for noninvasively and rapidly identifying those PC patients who could benefit from ICAM1 ADC therapy. In clinic practice, needle biopsies are commonly utilized prior to targeted therapy to examine the adequacy of target expression in tumor tissues however, but this approach is limited by its invasiveness and lack of accuracy (<50%) due to the intratumoral complexity and heterogeneity.^[^ ^32^ ^]^ To overcome these obstacles, we have developed a novel ICAM1‐targeting MRI probe and have used it to map the tumoral ICAM1 expression in an orthotopic PC model using MRI (**Figure** **4**A). We first engineered the ICAM1‐targeting MRI probe by covalently conjugating ICAM1 antibody with DTPA‐Gd, a clinically used MRI contrast agent.^[^ ^33^ ^]^ IgG‐Gd was prepared as a nontargeting control. ICAM1‐Gd or IgG‐Gd was i.v. administered into ICAM1‐expressing PANC‐1 tumor‐bearing mice at a dosage of 5 mg kg^−1^ mouse weight. At preinjection and 24 h postinjection of MRI probes, we performed in vivo MRI on PC tumor‐bearing mice with a set of MRI sequences, including T1, T2‐weighted spin echo imaging. In Figure 4B, we first analyzed high‐resolution T2‐weighted MRI images to locate PC tumors (yellow circle) in the peritoneal cavity. With the PC tumor located, we then used T1‐weighted MRI images to quantitatively measure MRI signal changes in the area of the PC tumor (yellow circle) as a function of intratumoral accumulation of gadolinium from administered MRI probes. In Figure 4C, the tumoral T1 MRI signal increased ≈50% in the ICAM1‐Gd group, while no MRI signal changes were observed in the non‐targeting IgG‐Gd group (*N* = 3 per group). These MRI signal changes were positively correlated with the level of antigen expression on the targeted tumors supporting the use of this approach to identify ICAM1‐positive patients who might benefit from ICAM1‐targeting ADC therapy.

## 3 Discussion

We have engineered a highly selective and potent ICAM1‐DM1 ADC, complemented by an MRI‐based molecular imaging modality that represents a novel precision therapeutic approach for PC therapy. We have demonstrated that by conjugating an anticancer drug with the active targeting ICAM1 antibody, we could effectively and specifically deliver this drug into PC tumors via ICAM1 antibody guidance while limiting the distribution to normal organs/tissues, which in turn, reduces off target toxicities. PC tumors in mouse models can be effectively suppressed by the ICAM1‐DM1 ADC treatment even with only two doses, resulting in significant inhibition on tumor growth and metastasis. Our therapeutic approach relies on the optimal efficient target that we identified for human PC based on an unbiased and quantitative screening. Additionally, noninvasive MRI provides a precision medicine approach for evaluating the accumulation and distribution of ICAM1‐DM1 ADC in the PC tumors, which helps to predict the therapeutic effect of our ADC.

Our data provide evidence that ICAM1‐ADC complemented by noninvasive MRI is a highly efficient targeted therapy for PC tumors. Although many approaches have been explored to improve the cytotoxic therapies that are typically limited by their dose‐dependent cytotoxicity, there are few being approved for the clinical applications, including albumin bound drugs, liposomal drugs, and antibody conjugated drugs. Compared with the albumin and liposomal formulations, which have the average size of around 100 nm, ADC is in the optimal size (≈5–15 nm) that is capable of escaping from reticularendothelial system (RES) uptake, resulting in a prolonged therapeutic window.^[^ ^34^, ^35^, ^36^, ^37^ ^]^ The blood half‐life is ≈4.5 days for ADCs, in comparison with 20–30 h of albumin and liposomal formulations.^[^ ^38^, ^39^, ^40^ ^]^ The prolonged circulation time as well as the conserved complex with bioactivities leads to more effective accumulation in the tumor site and less toxicity to the normal tissues. In contrast to albumin bound drugs, such as nab‐paclitaxel, also called Abraxane, has been found dissociate rapidly due to the weak affinity between albumin and paclitaxel resulting in a limited therapeutic index.^[^ ^34^ ^]^ Applying metronomic dosing may enhance antitumor activity,^[^ ^41^, ^42^ ^]^ for example, changing dosing every three weeks to dosing weekly, however, the lack of an efficient target that is specific for PC tumors still remains absent for these formulated drugs. Our strategy of adding an active targeting component to chemotherapeutics can significantly enhance their specific delivery efficiency to tumors.^[^ ^43^ ^]^

Our results demonstrate that ICAM1 is a novel ADC target for PC tumors due to its high tumoral overexpression and effective cell internalization. Since T‐DM1, an ADC that has been approved for solid tumor treatment, has shown its promising efficacy in breast cancer treatment, improving 4–7 months in overall breast cancer patient survival,^[^ ^44^, ^45^, ^46^ ^]^ multiple ADCs are under development for PC patients. However, to date, none of the targets that ADCs rely on to deliver the potent anticancer drugs has demonstrated sufficient and specific targeting capability in the clinic. For example, MEDI‐547, the ADC that developed for PC treatment with EphA2 targeting, failed in the clinic due to the severe side effect such as neuropathy.^[^ ^47^ ^]^ In comparison, ICAM1 is overexpressed at relatively higher levels in PC tumors than EphA2, ≈19 fold as that of the normal controls, while the average of EphA2 is about equal to the normal controls. The relatively higher expression levels of ICAM1 in PC tumors may render enhanced therapeutic effect and reduced side effects. The only targeted therapy for PC that has been approved by the FDA is the EGFR inhibitor, erlotinib. Although the combination of erlotinib and gemcitabine demonstrated improved survival, only a small subset of patients benefit from this target therapy because of the relatively low expression of EGFR in PC tumors. The expression level of ICAM1in PC cells is significantly higher than these targets that being tested in the clinic currently, including EphA2, EGFR, mesothelin, and transferring receptor. Recently ICAM1 was reported to be an efficient target in CAR‐T treatment of thyroid cancers, confirmed the specific targeting of ICAM1 with limited distribution in normal tissues.^[^ ^18^ ^]^ Furthermore, as shown in our studies, ICAM1 antibody also effectively suppresses PC cell migration in addition to the anticancer drug mertansine, DM1. Therefore, despite the need of long term safety studies in large animals before moving to clinical application, we anticipate that our ICAM1‐DM1 ADC may provide a synergistic effect for PC therapy.

The noninvasive MRI in our studies provides a clinically applicable diagnostic modality to our ICAM1‐DM1 ADC therapy, enabling us to bring precision therapy for PC patients. Conventional gene or biomarker profiling requires needle biopsies to confirm the specific biomarkers or mutant genes. However, gene profiling usually does not reflect the actual protein expression in situ.^[^ ^48^ ^]^ In addition, the biomarker evaluation from patient tissue arrays may not be accurate because of the heterogeneity of the PC tumor itself, where the stromal component may comprise up to 90% of the tumor mass.^[^ ^49^ ^]^ Although there might be an inevitable gap between preclinical models and real situation when moving to the clinic, MRI may serve as a reliable robust tool to reflect the distribution of the ADC in the whole tumor in three dimensions, as well as in the normal organs/tissues. We show that our strategy using noninvasive MRI to evaluate the tumoral accumulation of ICAM1 antibody is important in predicting the therapeutic effect and providing the noninvasive opportunity to titrate the treatment profile immediately.

## 4 Conclusion

The preclinical data described in this study demonstrate the effectiveness of our ICAM‐DM1 ADC in preclinical PC treatment, supporting the translational potential of its utilization in ICAM1‐overexpressing PC therapy. This study provides the basis for the initiation of large animal studies to evaluate the safety and efficacy of ICAM1 ADC in reparation for clinical trials. The utility of ICAM1 as a target can be extended to other advanced cancers and to other immunotherapeutics including CAR‐T cells or bi‐specific antibodies directed toward ICAM1. Moreover, this precision medicine approach can be potentially utilized for the targeted treatment of PC and other ICAM1‐overexpressing cancers, and improve the other underway early phase clinical trials that use tumor‐targeting therapeutics.

## 5 Experimental Section

##### Study Design

The goal of this study was to develop and validate a novel precision medicine approach for the treatment of pancreatic cancer. The studies were conducted in athymic nude mice to assess the therapeutic efficacy of ICAM1‐targeted ADC and the monitoring of targeted delivery. In vivo studies were conducted following a protocol approved by the Institutional Animal Care and Use Committee (IACUC) at Boston Children's Hospital. Sample size (*N* = 5–6 for treatment and *N* = 3 for MRI) was the standard for each experiment, providing sufficient statistically significant differences between different groups. Data was presented without any outlier exclusion. Mice were randomly divided into different groups and investigations were performed unblinded. Animal pain and distress was carefully monitored, and the tumor size was monitored once a week by IVIS. For the treatment study, the animals were sacrificed at the same terminal point when ascites were observed in several animals in the control group.

##### Quantification of Cell Surface Protein Expression

Pancreatic cancer cell and normal cell surface protein expression was evaluated by a BD FACSCalibur flow cytometer (BD Biosciences) as described previously.^[21‐23]^ Quantification of the protein density on the cell surface was determined with reference to quantum simply cellular microbeads, using the protocol as provided by the manufacturer. Briefly, 10^6^ cells were collected and rinsed twice through suspension–spin cycles. Cells were blocked by 1% bovine serum albumin (BSA) in PBS for 30 min in an ice bath. After BSA blockage, cells were incubated with PE conjugated antibodies for 1 h at room temperature, respectively. Cells were rinsed with 1% BSA in PBS three times, resuspended in PBS, and evaluated by flow cytometry.

##### Validation of ICAM1 Expression in Patient Samples

Immunohistochemical studies were conducted on paraffin‐embedded human PDAC and normal tissue microarrays (PA1002a, US Biomax). 80 human PDAC tissue and 20 human normal tissue microarray samples were evaluated for ICAM‐1 expression as described previously.^[21‐23]^ The individual tissue cores in the microarrays were scored by a gastrointestinal surgical pathologist, with no knowledge of sample identity. Immunostains were scored by calculating H‐scores in which the percent of cells staining strong (3+), moderate (2+), and weak (1+) were multiplied according to the formula: H‐score = 3× (% of cells staining 3+) + 2× (% of cells staining 2+) + 1× (% of cells staining 1+). Photomicrographs were taken on an Olympus BX41 microscope by using an Olympus Q‐Color5 digital camera (Olympus America Inc.).

##### Determining Antibody Targeting Efficiency on PC Cells

The in vitro specific binding of ICAM1 antibody to the human pancreatic cancer cell lines was assessed using PE‐ICAM1 antibody. IgG was used as the control. Cells were seeded in 8‐well chamber slides at a density of 5 × 10^3^ cells per well. After recovering for 24 h, the full media was replaced with that containing PE‐ICAM1 antibody, with 1% FBS. Cells were incubated with the PE‐ICAM1 antibody‐containing media at 37 °C for additional 4 h. The cell monolayer was then rinsed with cold PBS and fixed with 4% paraformaldehyde in the PBS solution. Cell nuclei were counterstained with 4′,6′‐diamidino‐2‐phenylindole hydrochloride (DAPI) using ProLong Gold Antifade Mountant. Fluorescence images were acquired and analyzed using a Zeiss LSM 880 confocal microscope.

In the imaging flow cytometry studies, cells were seeded in 6‐well chamber slides at a density of 1 × 10^6^ cells per well. After cell recovery for 24 h, the full media was replaced with that containing PE‐ICAM1 antibody, with 1% FBS. Cells were incubated with the PE‐ICAM1 antibody‐containing media at 37 °C for 1 h. The cell monolayer was then collected and rinsed with cold PBS twice, resuspended, and evaluated using an Amnis imagestreamX Mark II imaging flow cytometry (Luminex).

##### Preparation and Characterization of ADCs

IgG‐DM1 and ICAM1‐DM1 were prepared as described previously^[^ ^50^, ^51^, ^52^, ^53^, ^54^ ^]^ by mixing IgG or ICAM1 Ab (2.4 mg, 16 nmol) with SMCC‐DM1 (0.2 mg, 0.19 µmol) in a phosphate buffer (pH 7.2), rotating at room temperature for 1 h. Free SMCC‐DM1 was removed by Ultra‐4 Centrifugal Filter (30K MWCO). IgG‐DM1, ICAM1‐DM1 were washed with PBS (pH 7.4) for several times and redispersed in PBS. The ADCs were characterized by UV/vis spectroscopy and antibody drug ratios were calculated according to the equation

(1) $$\begin{array}{ccc} & & \mathrm{DAR}=C_{\mathrm{drug}}/C_{\mathrm{Ab}}=\left(A_{280}\varepsilon_{\lambda \left(D\right)\mathrm{mAb}}-A_{\lambda \left(D\right)}\varepsilon_{280\mathrm{mAb}}\right)\\ & & \left(A_{280}\varepsilon_{\lambda \left(D\right)\mathrm{drug}}-A_{\lambda \left(D\right)}\varepsilon_{280\mathrm{drug}}\right)\;/\;\left[\left(\varepsilon_{280\mathrm{drug}}\varepsilon_{\lambda \left(D\right)\mathrm{mAb}}-\varepsilon_{\lambda \left(D\right)\mathrm{drug}}\varepsilon_{280\mathrm{mAb}}\right)\right)\\ & & \left(\left(\varepsilon_{280\mathrm{mAb}}\varepsilon_{\lambda \left(D\right)\mathrm{drug}}-\varepsilon_{\lambda \left(D\right)\mathrm{mAb}}\varepsilon_{280\mathrm{drug}}\right)\right] \end{array}$$

IgG‐DTPA‐Gd, ICAM1‐DTPA‐Gd were prepared as reported previously.^[^ ^55^, ^56^ ^]^ Briefly, DTPAA (0.2 mg, 0.56 µmol) was slowly added to IgG or ICAM1 Ab (0.2 mg, 1.3 nmol) in NaHCO_3_ buffer (pH 9.0, 0.1 m), and the mixture was rotated at room temperature overnight. The DTPA‐conjugated IgG or ICAM1 Ab was purified by Ultra‐4 Centrifugal Filter (30K MWCO) and redispersed in citrate buffer (pH 6.5, 0.1 m). Then GdCl3 (0.1 mg, 0.27 µmol) in 0.1 m citrate buffer (pH 6.5) was mixed with DTPA‐conjugated IgG or ICAM1 antibody for 24 h rotated at room temperature. The free Gd^3+^ was removed by Ultra‐4 Centrifugal Filter (30K MWCO), and IgG‐ or ICAM1‐DTPA‐Gd was redispersed in PBS for subsequent use.

##### In Vitro Cytotoxicity Studies

Human pancreatic cancer cell lines were seeded in a 96‐well plate at a density of 5 × 10^3^ cells per well and allowed to adhere overnight. The culture medium was then replaced with medium containing free GEM, IgG, or ICAM1 conjugated DM1, Duo, MMAE, MMAF at different drug concentrations. After cells were cultured for another 72 h, the cytotoxicity was determined by CCK‐8 assay following the vendor‐provided protocol. Briefly, cells were carefully rinsed with PBS after the drug‐containing medium was removed, and this was followed by adding the CCK‐8 containing medium solution. The cells were then incubated with the CCK‐8 medium for 4 h. The plate was read at the absorbance wavelength of 450 nm using a microplate reader (Synergy2; BioTek). Cell viability was determined by comparing the absorbance of cells incubated with drugs to that of the control cells incubated without the presence of the drug.

##### In Vivo Treatment Effect in Orthotopic PDAC Mouse Models

All animal experiments were conducted following a protocol approved by the Institutional Animal Care and Use Committee (IACUC) at Boston Children's Hospital. PANC‐1 cells were transfected with a plasmid expressing the luciferase and GFP genes according to the manufacturer's instructions (MGH, Boston). Successful gene transfer was confirmed 72 h after infection, by the visualization of GFP on fluorescence microscopy. Stably transfected cells were sorted twice by flow cytometry for GFP signal using a FACSAria II flow cytometry (BD Biosciences), and maintained in DMEM‐10% FBS. The orthotopic pancreatic cancer model was prepared by injecting PANC‐1 cells into the pancreas of 8‐week‐old male athymic nude mice (Charles River) using an established surgical method.^[^ ^57^, ^58^ ^]^ Mice were anesthetized by isofluorane (5% with O_2_) during the surgery. Incision was made on the left flank of the abdominal region where the pancreas was typically located, behind the middle of the spleen. The pancreas was then gently pulled out using forceps and 50 µL 1 × 10^6^ PANC‐1 cells were carefully injected into the pancreas. After injection, the pancreas was placed back in the abdominal cavity before the abdominal muscle and the skin were closed with 4‐0 polysorb sutures and surgical staples. Treatment was started after two weeks of recovery. Animals were randomly divided into four groups (*N* = 6): PBS control, treated with GEM, treated with non‐targeted IgG‐DM1, and treated with ICAM1‐DM1. The mice were treated through intravenous tail vein injection at a dose of 15 mg kg^−1^ mouse weight per three weeks for IgG‐DM1 and ICAM1‐DM1, 5 mg kg^−1^ mouse weight twice a week for GEM, while the control group received only PBS injection. In total, there were two injections with 3‐week intervals for ADC treated and control groups, and 12 injections with 3‐ or 4‐day intervals for GEM. The body weights were measured twice a week, and tumor growth was monitored using the IVIS Spectrum Imaging System (PerkinElmer) after mice received i.p. injection of D‐luciferin.

##### In Vivo Tumor Targeting and Distribution

In vivo MRI was performed on the tumor‐bearing mice in two groups (*N* = 3), which were injected i.v. with IgG‐Gd and ICAM1‐Gd (at the dosage of 5 mg kg^−1^ mouse weight), respectively. Images were obtained at preinjection and 24 h postinjection with a 9.4 T Bruker Horizontal Bore MRI with turbo spin echo sequence for T1‐ and T2‐weighted MRI. The imaging parameters were as follows: repetition time (TR) of 1,523 ms, TE of 33 ms, 340 × 220 matrix, 40 × 28 mm^2^ field of view, 180° flip angle, and 0.6 mm slice thickness for T2‐weighted imaging; TR of 700 ms and TE of 22 ms for T1‐weighted imaging. To quantify the signal intensity for tumor, regions of interest (ROIs) were drawn around the whole tumor at the same slice with the same imaging depth. The pixel intensity was calculated and normalized to the area of ROIs by ImageJ software.

##### Statistical Analysis

For in vitro studies, *N* = 5 in each group and the experiment was repeated three times unless identified specifically. For in vivo studies, *N* = 5,6 per group for treatment and *N* = 3 per group for MRI. Quantitative data are presented as means ± SD. Differences were compared using an unpaired *t*‐test or One‐way ANOVA. Statistics were performed using Microsoft Excel software or Prism (Graph Pad Software Inc.). *p* values ≤0.05 were considered statistically significant. All data associated with this study are available in the main text or the Supporting Information.

## Author Contributions

J.H., P.G., M.A.M. all contributed key ideas, designed the experiments, and analyzed the data. A.T.A. performed IHC staining of patient TMA and analyzed data. J.H. and P.G. performed the in vivo treatment studies and imaging. J.H. performed the FACS screening, IF staining, QPI experiments, ADC, and MRI probe conjugation, in vitro cytotoxicity studies, and ex vivo tumor and organ analysis. P.G. performed the imaging flow cytometry. J.H., P.G., M.A.M. all contributed to the writing of the manuscript. The results presented in the paper have been included in the filing of U.S., Patent Application Serial No. 62/891170.

## Conflict of Interest

The authors declare no conflict of interest.

## Supporting information

Supporting Information
