# Supplementary material for: A Rationally Designed ICAM1 Antibody Drug Conjugate for Pancreatic Cancer
Source: Adv Sci (Weinh). 2020 Nov 3;7(24):2002852. doi: 10.1002/advs.202002852 (PMC7740099; doi:10.1002/advs.202002852)

WILEY-VCH

© 2020 Wiley-VCH GmbH

ADVANCED SCIENCE
Open Access

Supporting Information

for *Adv. Sci.*, DOI: 10.1002/advs.202002852

A rationally Designed ICAM1 Antibody Drug Conjugate for Pancreatic Cancer

Jing Huang, Agoston T. Agoston, Peng Guo, Marsha A. Moses**

Supporting Information

A rationally designed ICAM1 antibody drug conjugate for pancreatic cancer

Jing Huang, Agoston T. Agoston, Peng Guo, Marsha A. Moses**

Supplementary Materials

Materials

Purified anti-human CD54 Antibody (Clone:HCD54), phycoerythrin (PE)-conjugated mouse anti-human ICAM-1 antibody (PE-ICAM1) and PE conjugated mouse IgG isotype (PE-IgG) were purchased from BioLegend(San Diego, CA, USA). ADC prescreening G-DM1, G-MMAE, G-MMAF, G-Duoca were purchased from Levena Biopharma (San Diego, CA). SMCC-DM1 was purchased from Medkoo(Morrisville, NC, USA). Zeba™ Spin Desalting Columns, (7K MWCO), Alexa Fluor 647 NHS ester, the Lab-Tek II Chamber Slide System, ProLong Gold Antifade Mountant was obtained from Thermo Fisher Scientific. Gemcitabine hydrochloride (GEM), gadolinium(III) chloride hexahydrate ($GdCl_3 \cdot 6H_2O$), diethylenetriamine-pentaacetic dianhydride (DTPAA), sodium bicarbonate, sodium citrate tribasic dihydrate were purchased from Sigma-Aldrich (St. Louis, MO). Dulbecco's PBS, DAPI, Quant-iT RNA Assay Kit, 0.25% trypsin/2.6 mM EDTA solution, Gibco DMEM, Gibco DMEM/F12(1:1), Roswell Park Memorial Institute (RPMI)-1640 medium, and McCoy's 5A medium were purchased from Invitrogen (Carlsbad, CA). MEGM Mammary Epithelial Cell Growth Medium was purchased from Lonza (Basel, Switzerland). Quantum Simply Cellular microbeads were purchased from Bangs Laboratory (Fishers, IN). The Dojindo cell counting kit CCK-8 was purchased from Dojindo Molecular Technologies (Rockville, MD, USA). Human pancreatic cancer tissue and normal tissue arrays (PA1002a) were purchased from US Biomax (Derwood, MD).

Cell Culture

PANC-1, BxPC-3, Capan-1, Capan-2 and HPNE cells were purchased from ATCC (Manassas, VA). HPDE cells were purchased from Kerafast (Boston, MA). PANC-1, Dulbecco's Modified Eagle's Medium with 10% FBS, BxPC-3, RPMI-1640 Medium with 10% FBS; Capan-1, Iscove's Modified Dulbecco's Medium with 20% FBS, Capan-2, Modified McCoy's 5a Medium with 10% FBS; HPNE, 75% DMEM without glucose with additional 2 mM L-glutamine and 1.5 g/L sodium bicarbonate, 25% Medium M3 Base, FBS 5%, 10 ng/ml human recombinant EGF, 5.5 mM D-glucose (1 g/L), 750 ng/ml puromycin; HPDE, Keratinocyte Basal Medium with supplied supplements (Lonza, Clonetics KBM). All cells were maintained at 37 °C in a humidified incubator with 5% (*vol/vol*) CO_2.

Therapeutic effect of ICAM-1 Ab

The *in vitro* therapeutic effect of ICAM1 antibody to the human pancreatic cancer cell lines was assessed using quantitative phase imaging. IgG was used as the control. Cells were seeded in a 6-well plate at a density of $5×10^4$ cells/well. After recovering for 24 hours, the full media was replaced with that containing ICAM1 antibody or IgG at a dosage of 2 μg/mL. Cells were incubated with the ICAM1- or IgG-containing media at 37 °C for 24 hours. After that, the plate was placed under a quantitative phase imaging microscope (Holomonitor M4, Phase Holographic Imaging Phi AB, Lund, Sweden) setting in an incubator and imaged for an additional 24 hours with a 5 min interval. Cell motion, morphology and proliferation were then analyzed using Hstutio4.

Histology

The organs (liver, spleen, kidney, pancreas, heart, lung and muscle) and tumor samples were collected at the end point. Pathologies of orthotopic PANC-1 tumors treated with ICAM-DM1, IgG-DM1, GEM and PBS were investigated by H&E staining, Ki67

staining, and ICAM1 immunohistological staining. All staining was performed for the tumor slices following the standard protocol.

Figure S1. Metastasis in mouse organs after treatment. Representative H&E staining of mouse organs from different treatment groups. Scale bar, 100 μm. Micrometastasis sites were indicated by yellow asterisks. Increased lymphocytes in spleen were indicated by red asterisks.

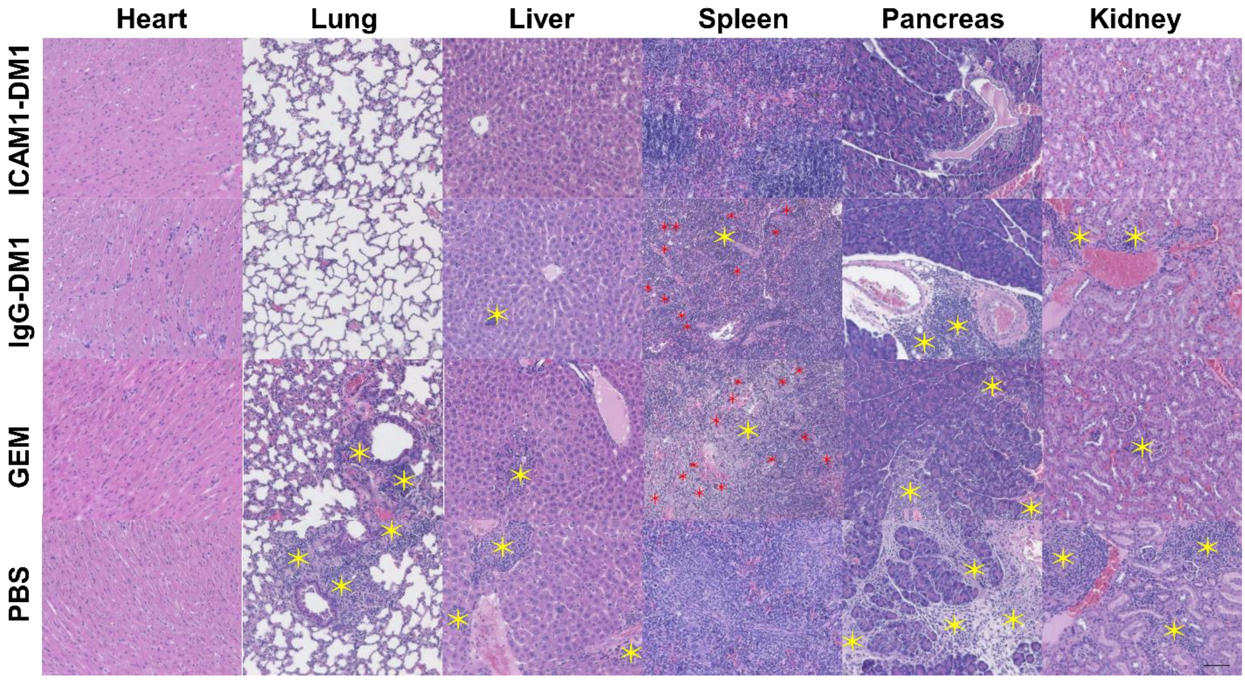

Supplement: Supplementary file 1 — Supporting Information [file ADVS-7-2002852-s001.pdf]
